# The transformative journey of community health workers in implementing a lifestyle intervention in Brazil: A qualitative study

**DOI:** 10.1017/cts.2024.612

**Published:** 2024-10-10

**Authors:** Andiara Schwingel, Ana Selzer, Deanivea Mendes Felix, Wojtek Chodzko-Zajko, Daniel Umpierre, Felipe Reichert, Pedro Hallal

**Affiliations:** 1 University of Illinois Urbana-Champaign, Urbana, IL, USA; 2 Federal University of Rio Grande do Sul, Porto Alegre, Brazil; 3 Federal University of Pelotas, Pelotas, Brazil

**Keywords:** Community health workers, healthy lifestyles, physical activity, eating habits, mindfulness, intervention implementation, healthcare model

## Abstract

**Introduction:**

Community health workers (CHWs) stand as critical frontline agents within the Brazilian healthcare system. In this qualitative study, we examined the impact of a community-based behavioral change intervention spearheaded by CHWs.

**Methods:**

The intervention focused on promoting healthy behaviors – physical activity, nutrition, and emotional well-being – among individuals aged 50 and older living in a rural community in Brazil. The intervention was designed, implemented, and evaluated in close collaboration with CHWs and local administrators. The implementation of the intervention unfolded in two waves, each lasting 12 months. Interviews with CHWs, health administrators, and intervention participants conducted at post-intervention and 6-year follow-up centered on CHWs as delivery agents and examined the implementation of the intervention in primary care contexts around adoption, implementation, and long-term maintenance.

**Results:**

Inductive analysis revealed four themes that highlight CHWs’ motivation to take active roles in health promotion and overcoming challenges such as unfamiliarity with new roles or limited training. In addition, enhanced community bonds, job satisfaction, and trust in CHWs gained through the intervention, empowered CHWs to realize their potential and importance. Another important area relates to the CHWs’ ability to leverage their deep community ties and cultural insights to enhance the intervention’s significance. CHWs’ participation in the program also led to personal benefits and self-care practices, setting an example for the community they serve.

**Conclusions:**

This study underscores the positive impact of a community-based intervention led by CHWs. Such programs have the potential for nationwide dissemination, leveraging the CHWs’ widespread presence and deep community integration.

## Introduction

Advancing public health requires bringing community resources together and changing the systems that touch people’s everyday lives. This is especially critical in the prevention and management of chronic diseases, which are the leading causes of death and disability in Brazil and globally, and impose significant financial burden on the public health system due to the high costs of management. Physical activity, a balanced diet, and mindfulness are widely recognized as modifiable behaviors associated with a variety of health outcomes, including obesity, cardiovascular diseases, diabetes mellitus, cancer, hypertension, depression, and effects on functional ability, independence, and well-being [1,2]. Unhealthy lifestyle practices are widespread within the Brazilian society. Data from the 2019 VIGITEL surveillance system showed that between 15 and 22% of adults living in Brazilian state capitals had obesity, and 50–65% were physically inactive in leisure time [3]. A situation that has only worsened with the COVID-19 pandemic, resulting in an increase in these risk factors [4].

A notable initiative sponsored by the Brazilian Government is the Family Health Strategy (ESF) [5]. Since its inception in 1994, the ESF has been the cornerstone of Brazil’s public health efforts within primary health care, aiming to deliver comprehensive healthcare to families in their homes, clinics, and hospitals. About 40,000 Family Health teams operate across nearly all of Brazil’s 5,560 municipalities, serving up to 1,000 families or 4,000 individuals each, and covering 62.6% of the population (about 131.2 million people) [6,7]. These teams comprise doctors, nurses, dentists, and community health workers (CHWs), among other health professionals. Although the focus has predominantly been on primary clinical care, there is a growing interest in promoting healthy lifestyles within communities. For example, research by Benedetti *et al*. [8] on the role of primary care in delivering physical activity programs to older adults in Brazil confirms the potential for implementing and disseminating behavior change and physical activity programs in local public health centers. Silva *et al*. [9] published a systematic review of studies, including some from Brazil, on the implementation determinants of physical activity interventions in primary health care settings. Their study, examining assessment, counseling, prescription, and referral, suggested that the knowledge and skills of involved health professionals are particularly relevant factors in designing interventions.

The success of implementing community-based health programs often rests in the hands of local leaders. CHWs have been essential members of healthcare teams since 1991, with 265,000 working across the country [10]. CHWs’ primary responsibilities include care practices, surveillance, health education, health communication, administrative practices, intersectoral coordination, and social mobilization [11,12]. CHWs play a crucial role in health initiatives due to their extensive reach and cultural humility, which enable them to tailor interventions to community needs [13]. Previous studies have indicated that health-related content can be challenging for certain groups, such as the elderly and those with limited formal education. Integrating personally meaningful information can enhance the effectiveness of community-based health programs [14,15]. Being members of the communities they serve, not only aids in cultural understanding and identification of barriers but also helps in building trust. This approach is especially critical in rural and low-income areas, where CHWs are instrumental in connecting individuals to primary care services and disseminating health information. CHWs advance health equity and serve as cultural mediators, helping healthcare providers understand the cultural and social contexts of their patients’ lives [16]. CHWs are important in primary healthcare teams as they can help to alleviate the workload faced by providers [17]. As providers focus on clinical treatments, CHWs bring an opportunity to promote chronic disease self-management and reduce the reliance on medication alone [18]. CHWs often engage in long-term, relationship-driven work with clients [19]. Their social embeddedness within the communities they serve is paramount to fostering sustainable health practices.

The implementation of community-based health behavior interventions led by CHWs represents an under-explored area of research in Brazil, addressing a notable gap in the current literature. To address this shortcoming and inform the implementation science literature, this qualitative study focuses on CHWs as delivery agents and examines the primary care contexts around adoption, implementation, and long-term maintenance [20]. Details of the intervention protocol and the associated implementation challenges have been published elsewhere. We framed this study within the context of designing for dissemination, using the Reach, Effectiveness, Adoption, Implementation, and Maintenance (RE-AIM) framework [21]. As an implementation science framework, RE-AIM has evolved over its two decades of application [22], presenting an opportunity to examine the complex layers of a community-based behavioral change intervention spearheaded by CHWs. Leveraging Brazil’s public health infrastructure, the study advocates for maximizing reach with a shift in the care model to place CHWs at the forefront of promoting healthy lifestyles.

## Methods

This study was conducted in a rural city in the State of Rio Grande do Sul, Brazil, with approximately 3,600 residents, and 26.5% of its population aged 50 and older. The state has the sixth-largest population in Brazil, with over 10 million inhabitants and a significant proportion of the population of Italian and German descent. It is slightly more developed than most parts of Brazil, boasting a literacy rate of 97%, one of the highest in the country. The city of Dona Francisca falls in the middle of the state profile with a Human Development Index of 0.697. Primary healthcare covers 100% of the population, a common trend for small cities in the state [23,24]. This was the first large epidemiological study conducted in the city. Partnerships with the Municipality’s Public Health Department, public health center’s personnel, and CHWs were established prior to the start of the intervention.

This article focuses on qualitative data from a larger study that also assessed quantitative data from participants of the intervention. To add context, approximately 900 older adults between 50 and 80 years of age resided in the city at the time of the study, and all were invited to participate in the study during CHWs home visits. The study included 649 community-dwelling older adults who self-reported one or more chronic conditions and had not participated in an exercise program for the past six months. Those with cognitive decline or inability to follow study protocols were excluded. Conducted as a randomized controlled trial, we used cluster randomization by neighborhood to reduce the risk of contamination and account for contextual effects. The city was divided into eight areas, each assisted by one of the eight CHWs. In year one (2015–2016), 381 eligible residents of the four areas selected for the experimental group joined the intervention, whereas 247 residents of the remaining four areas joined a waiting list (control group) and participated in evaluation only. The waitlist group received the full program in year two (2016–2017) of the intervention.

This culturally tailored program consisted of a behavioral change intervention focused on physical activity, eating habits, and mindfulness, adapted from previously published evidence-based programs [25,26]. The intervention used a participatory approach and collaborated with CHWs and local leaders (e.g. health professionals and priests) to design a program that meets the needs of the community. With their help, the intervention content was culturally adjusted to the community they serve and are part of. The program comprises six group workshops, each lasting approximately 2 hours. Details about the content of these workshops are outlined in Table 1. Participants received a toolkit containing booklets and gifts related to the workshop. Additionally, the program employs behavior change-based tools, including action plans, personalized self-management tips, and friendly reminders. In addition to the workshops, information included in these sessions was often reinforced by CHWs in informal conversations that occurred between them and their clients during the course of house visits and other incidental contacts. Further details on the intervention protocol and implementation can be found elsewhere [20].

**Table 1.** Educational group workshops content

Workshops	Content
Get energized with healthier eating habits	Eat plenty of fruits and vegetables Prefer whole grains The risks of sugar and where sugar can be hidden Drink water
Live an active lifestyle your way	Benefits of aerobic physical activity All types of movement are important Guidelines on physical activity Step count
Feeling good about life	Mindfulness benefits and exercises Love yourself Practice to smile more Cultivate calmness Mindfulness on everyday tasks
Healthy meals for your health and well-being	The risk of the excessive consumption of salt and fat How to buy healthier versions of food items – reading labels How to cook healthier meals
Strong for life	Benefits of resistance training Full-body exercise suggestions Guidelines on resistance training
Improving your quality of life	Understanding reactions to experiences Mindfulness practices Mindful exercising Loving consciousness

The core of the intervention centered on CHWs, who served as participant recruiters, received training, delivered the program, and assisted with the evaluation. All eight CHWs working in the city were recruited and participated in this study (their demographics are described in Table 2). CHWs were randomly assigned to receive training in either year one (n = 4) or year two (n = 4), according to the study design previously described. The research team provided training through a hybrid format that combined in-person and virtual sessions. The training spanned 10 days, with each session lasting 4 hours and covering various program elements, essential health information, and workshop role-playing activities. To ensure comprehensive understanding, the training included quiz checks and feedback loops to reinforce concepts not initially well grasped. Training was customized for each CHW, allowing for necessary corrections, adjustments, and improvements in program delivery. To minimize barriers, CHWs were given access to computers and tablets, printed program manuals with curricula, and guides for each program component. Throughout the intervention, the research team supported the CHWs, helping them to address and overcome any challenges that arose.

**Table 2.** Demographic characteristics of community health workers at post-intervention

		Total (*N* = 8)
Age, mean (SD)		39 (13)
Women, *N* (%)		7 (87.5%)
Race		
White		5 (62.5%)
Brown		3 (37.5%)
Education, *N* (%)		
High school or less		6 (75%)
More than high school		2 (25%)

Semi-structured interviews were conducted with a subsample of program participants (n = 37), administrators (n = 7), and all CHWs (n = 8) post-intervention in 2016 and 2017. An additional follow-up assessment was conducted at the 6-year maintenance point in 2023, with a subset of CHWs (n = 6), administrators (n = 4), and former program participants (n = 29). The interview guides were developed using the RE-AIM framework, specifically focusing on the domains of adoption, implementation, and maintenance. These domains were examined from the perspectives of the short-term (around the intervention period) and the long-term (6-year follow-up). Three trained researchers conducted the interviews. Data was voice-recorded and transcribed verbatim. Themed analysis was conducted using the Nvivo 14 software by two independent researchers, with help of the third coder when agreement was not reached. Data was coded inductively in Portuguese, with frequent meetings for code agreement and later aggregated into themes to make sense of the data. The study was reviewed and approved by an ethics committee overseen by the Ministry of Education through the Federal University of Pelotas, ensuring adherence to ethical standards for research involving human subjects (protocol 797.437).

## Results

The findings showed that the implementation of a behavioral change program resulted in a shift in the roles and perceptions of CHWs, challenged conventional healthcare paradigms, derived benefits from a participatory approach, and encouraged self-care practices among CHWs. The four themes that emerged from the analysis are described below.

### Theme 1: Pushing the Boundaries and Redefining the Care Model

Initially, the behavioral change program was unfamiliar to the healthcare team, as it diverged from their traditional training and roles. Healthcare professionals, including CHWs and local administrators alike, perceived the program as innovative, yet were uncertain about its potential impact and the community’s response. Key challenges included granting CHWs more autonomy – moving away from their roles as mere assistants to autonomous health professionals during group activities, the additional workload, and changing community perceptions regarding their lead roles in delivering the program. This uncertainty stemmed from previous resistance to new activities, causing some to doubt the CHWs’ commitment to the program. CHWs expressed initial concerns about their competency, experience, and ability to independently manage the program, feeling insecure about diverging from their routine tasks. The following quotes illustrate some of the initial sentiments expressed by CHWs.

*We were feeling nervous about offering the program. We would say “where are the researchers who developed the program? No one here to help? Is it all on us to run the program?” Earlier that day I went to see my boss and shared “I am not feeling well, I think my [blood] pressure is low. We have the program today. I think I need some medication to calm my nerves.” [CHW]*

As shown below, it became evident that as CHWs overcame initial challenges, their confidence increased after a few sessions, resulting in the delivery of the program no longer being perceived as a stressor.

*I overcame my fears. I used to feel nervous to speak in front of everyone. We [CHWs] always tried to pass the turn to the other. But in the past workshops, I took the lead without problems. [CHW]*

*After this project, I can distinguish between the old and the new me. For me, speaking in public was the end of the world, but I had to do it… I learned in so many ways. It was really interesting. [CHW]*

This perspective was further validated by administrators who worked closely with the CHWs, as described in this quote:

*The growth of the CHWs is amazing. In the beginning I thought that they were resistant to the program. When they would come to me feeling insecure and worried that it would be too much for them, I would encourage them and say “try, this will be a great experience.” Today we see them very different, completely different. They are more engaged and knowledgeable. [Administrator]*

The program was instrumental in enhancing CHWs’ knowledge and reshaping their approach to healthcare, shifting home visit focus from solely diseases and medication to also emphasizing prevention through healthy eating and physical activity. This marked a significant change in mindset from prioritizing illness treatment to promoting overall health.

*We are talking more about nutrition. Before [the program], our home visits were more focused on medication. We are now talking about physical activity… we are taking the focus off medication. [CHW]*

*In this program, we’re promoting health. We don’t work just with people who have a condition, we work with both. At home visits, you look at them and assume that because they’re healthy, you do not have to do anything. It doesn’t mean that they know everything. You have to take care of them for them to keep being healthy, for them not to get sick. So, I think this is a great program that does not focus just on a chronic disease… we have to prevent them to developing one, that’s why it is open to the population. [CHW]*

The program also addressed and began to dismantle the preconceived limitations of CHWs’ roles. Initially, some community members expected physicians to lead the workshops, underestimating CHWs’ capabilities in health education. The quote below illustrates the shift in these perceptions, showcasing the CHWs’ potential in leading health promotion efforts.

*The first thing clients would ask was who will give the workshop. “Will it be a doctor?” I would say: “No, we [CHWs] are being trained and will lead the workshop.” I am not sure what they expected, but because they see doctors as God here, they may have expected someone else. Now clients trust in us and feel comfortable with what we are doing in the program. [CHW]*

*They’re advising them [population] very nicely. For instance, the other day, I went to the park and there was this lady that participates in the program and she was walking. It is so nice to see people being active! [Administrator]*

*The CHWs have such a great way of explaining things to us… That day, they gave a presentation on fruits. Even at my age, I didn’t know that information. I always say, “the meetings will not be in vain’; there’s always something new to learn. [program participant]*

### Theme 2: Recognition, Respect, and Empowerment

As a result of participating in the implementation of this program, CHWs reported feeling recognized and valued by both administrators and the community, noting a departure from the previous physician-centered model. The quotes below illustrate the feelings of increased recognition and visibility:

*We stopped focusing only on supporting the doctor or nurse. We are used to work behind them. The doctor goes first, talks to patient, and you [CHW] stay behind. So, with the program, we were on the frontline. CHWs themselves working. CHWs being the face [of the program] and showing leadership. We can make a difference. [CHW]*

*I feel useful, this is very rewarding. I feel that we [CHWs] are helpful to the community. I feel that they are recognizing our work, I am feeling satisfied about my work. [CHW]*

*We were happy because we realized that our work was being well received, right? [CHW]*

*CHWs are so dedicated, I observe them, they are everywhere, in the heat. [CHW’s name] was sick and she came to remind me about the program. I can see that they are doing their best to help us. [program participant]*

The program not only enhanced recognition and visibility but also fostered a sense of empowerment among CHWs, strengthening their sense of identity both individually and as a collective. This empowerment led to improved teamwork and communication, encouraging CHWs to collaborate more closely. They learned to identify and leverage their unique strengths and address weaknesses when distributing tasks, as evidenced by the following quotes:

*We worked together for many years, 5 years, but we didn’t talk much with each other. Now we started talking and it has been great for the group. Now we are better colleagues. Before [the program] we didn’t meet much. CHWs are now a team, we call each other, we say “let’s study,” “let’s prepare the activities for the project” [CHW]*

*At the end of the first workshop, we hugged each other. We got emotional celebrating that we did it. We couldn’t believe it. [CHW]*

*Before [the program] it was very different, everyone [CHWs] would work on their own things, we would meet each other at a group meeting, that’s it. [CHW]*

This acknowledgment of empowerment and recognition underscores the program’s success in redefining CHWs’ roles, indicating a shift toward a more inclusive and participatory healthcare model that values the contributions of CHWs in disease prevention and management. This sentiment is highlighted below.

*Through this program, CHWs gained respect, from the health department and the mayor’s office. They are supporting and encouraging CHWs to lead more community initiatives. [Administrator]*

*They [CHW] have a strong bond, and the population trusts them a lot. People open up to them during home visits. They help when needed, are always available… their work is seeing how they are, the health of the municipality. [Administrator]*

### Theme 3: the Centrality of Culture in Enhancing Intervention Significance

CHWs played a pivotal role in customizing the program to meet the needs of the population, thanks to their prolonged engagement with and deep integration into the community. Their intimate understanding of what resonates with participants allowed them to dynamically adapt the program. CHWs enhanced the program’s appeal by modifying language, integrating resources such as videos and interactive activities, and incorporating examples drawn from their own experiences and the community’s context. The following quotes offer illustrations of this perspective.

*In our community people eat a lot of starches, like bread, pastries, cookies. I think the materials should list these examples to explain the amount of sugar in them. On our own, we researched these foods to use as examples. I had cookies and bread at home and brought them to the workshop when talking about carbohydrates. [CHW]*

*We [CHWs] adjusted some information. We needed to revise some information because clients would not understand otherwise. We [CHWs] discussed some program activities and agreed on adjustments to make them more interactive. Also, we wanted to provide more examples about ourselves, things that happen every day in our community. That way the activities were easier to understand and to teach. [CHW]*

These adjustments not only made the material more relatable and easier to teach but also underscored the critical importance of involving CHWs to ensure engagement and retention. Their ability to tailor the program in real time demonstrates the value of their insights in making health interventions more culturally sensitive, engaging, and effective. The quotes below provide illustrations of these points.

*The way the information is described here seems strange. People will not understand. Many people cannot read well and they will have problems understanding descriptions like this one about “…food portion and quantity.” This can affect participation in the program. [CHW]*

*We think the training manual could be simplified. There is too much information here that we do not use, somethings are repetitive. I think it should be shortened to focus on what we need to say. I think the rest of the material is good. The booklets to clients are nicely done. People shared some issues about the research part. I had to be with some clients during the evaluation. Certain aspects, some words, they didn’t know the meaning. There were questions they didn’t understand and they would say “[CHW’s name] stay with us.” If I had already left their home, they would call me back. [CHW]*

*“They know us. I live there, they already know me. Sometimes they say, oh, good, you came today, I needed to talk.” [CHW]*

### Theme 4: Walking the Walk: Self-Care Matters

CHW recognized significant personal benefits from engaging in the program they facilitated, underscoring their dual role as educators and community members with similar health education needs as the participants. As illustrated in the quotes below, their involvement in delivering the program enabled them to grasp and apply the same health principles to their own lives.

*I changed a lot. Especially about my health. I lost 14 kg because of the activities we did. I changed a lot in relation to sweets and soda. [CHW]*

*I really liked the program because I am a sedentary person and didn’t use to do exercise. Then I finally got it, you know. I got that exercise is very important, all of us should do… We should start slow, but should exercise. [CHW]*

This dual engagement highlights the unique position of CHWs, who not only contribute to the community’s well-being but also enhance their own health and lifestyle through the knowledge conveyed by the program. This process of learning and self-application reinforces the symbiotic relationship between teaching health concepts and embodying them, thereby enhancing CHWs’ effectiveness, credibility, and fostering a culture of health within the community. The following quotes illustrate these observations.

*I changed a lot, emotionally and professionally. I learned to be a better listener, even when I am stressed out. The program taught me to reflect on my own behavior and I learned to calm down, count until three, think before speaking. This was one of the main lessons for me. [CHW]*

*I cannot express with words. I feel another person. Everything changed, from physical health, eating habits, to how I approach my clients in their homes. To be able to encourage people is so fulfilling. [CHW]*

*I started looking into myself, reflecting, and paying more attention to my life. I realized that I was spending most of my time caring for my family and house, and I wasn’t being active. Walking, I now started walking. I know this benefits my health. I also focus more on my social life now. I take my time talking and visiting people. I know this makes me a better person and helps others be better too. [CHW]*

## Discussion

Our findings reveal important insights into the transformative journey of CHWs in implementing a lifestyle intervention that initially faced unfamiliarity and skepticism among health professionals, administrators, and CHWs themselves. This was in part caused by the program’s deviation from traditional healthcare approaches and the adding responsibilities to the conventional roles played by CHWs. The intervention not only enhanced the CHWs’ skills and knowledge, particularly in promoting healthy lifestyles, but also significantly altered their approach to healthcare, moving from a focus on primarily disease and medication to one that added healthy lifestyles and self-care. The concept of challenging conventional care models is not without precedent. For instance, Pimentel *et al*. [27] examined the integration of mental health promotion within primary healthcare in Brazil, highlighting the country’s unique position to spearhead innovations in health promotion, prevention, and care. They further proposed that CHWs could serve as a pivotal platform for extending these efforts beyond primary care clinics to achieve wider population reach. Costa *et al*. [28] reviewed global literature on physical activity interventions led by CHWs, showcasing their important contribution in disease prevention and health promotion, particularly in promoting physical activity. Additionally, Schaaf *et al*. [29] discussed the varied roles of CHWs, conceptualizing their function on a spectrum that ranges from service extenders to social change agents. Here, service extenders are seen as enhancing the existing health system’s reach, whereas social change agents are portrayed as instrumental in driving systemic health changes and addressing broader social determinants of health.

The intervention took place in a rural area with a population density similar to other cities with under 5,000 inhabitants in Brazil. The last national census showed that the majority (95%) of Brazilian municipalities have under 20,000 inhabitants, indicating that Brazil is predominantly a country of small towns and cities [30]. Rural areas often face significant challenges in healthcare delivery due to geographic isolation, limited infrastructure, and resource scarcity, while urban areas grapple with issues related to higher population density and greater socioeconomic disparities [31]. Although both rural and urban settings have unique challenges, the complexity and barriers associated with rural healthcare delivery can make it uniquely challenging to work with populations in these areas [32]. CHWs in rural areas are crucial as they often serve as the first point of contact with the healthcare system and the strongest link between people and primary health care. Their social embeddedness in the communities they serve allows them to tailor health interventions to sub-populations experiencing a high burden of disease. The training and empowerment of CHWs to promote behavior change is essential, particularly in rural areas where access to training and education may be lower. A study on breast cancer demonstrated that CHWs meet health needs and promote screenings, leading to improved health outcomes in rural settings [33]. The effectiveness of CHWs is linked to their acceptance and trust within the community. This trust enables them to address health and social concerns effectively, which is particularly important in rural areas where residents might have less frequent interactions with formal healthcare providers. The present study did not focus on urban settings where the intervention would require significant adjustments due to profound differences. For instance, regarding physical activity, urban residents have more opportunities for leisure-time physical activity, while rural residents engage more in work-based physical activity, reflecting the distinct lifestyle and occupational demands in rural areas [34].

Since 1991, the profession of CHWs in Brazil has become increasingly integrated into the health care system. Krieger *et al*. [32] examined the integration of CHW practices into the Brazilian healthcare system and the effects of this institutionalization. They describe that CHW roles fall into various categories of health promotion and disease prevention. However, recent legislation impacting CHWs has led to a noticeable decline in time allocated for health promotion activities. Krieger et al. also note that certain prevention tasks are only assigned to CHWs who have completed the necessary technical training and are supported by clinically trained team members. Our research suggests the possibility of an important shift in community perceptions of CHWs, from expectations of physician-led interventions to a recognition and acceptance of CHWs as qualified health educators and leaders in health promotion. This change signifies a broader shift in the care model, where CHWs are seen as integral to the healthcare team and not merely as shadows behind other health professionals. The program’s success in redefining the roles and perceptions of CHWs underscores the potential for innovative interventions to break down traditional healthcare hierarchies and promote community trust in CHWs. A milestone was reached recently when CHWs were formally recognized as healthcare workers by the Health Ministry, marking a historic moment in Brazil’s healthcare landscape [10].

Central to this transformation was the intervention’s role in fostering a robust sense of identity and solidarity among CHWs, facilitating a collaborative environment that empowered them to leverage their collective strengths and assume leadership roles. This empowerment was further exemplified by their proactive engagement, underscoring a shift in community dynamics wherein CHWs are now seen as active health promoters. This contributes to an evolution in CHWs’ professional status and self-perception, driving a profound impact on their engagement, knowledge, and overall job satisfaction. Empowering CHWs has significant implications for the future of healthcare delivery, particularly in resource-limited settings where the presence and involvement of CHWs can bridge critical gaps in access to care and health behaviors. By highlighting the transformative journey of CHWs in our study, we underscore the imperative for continued investment in initiatives that elevate the role of CHWs. This paradigm shift not only enriches the professional lives of CHWs but has the potential to yield substantial improvements in community health outcomes. Our findings demonstrate the critical need for health policies and programs that acknowledge, support, and harness the unique roles and potentials of CHWs in the broader public health framework. Glenton and collaborators [35] stress the importance of involving CHWs in the co-design of new roles and tasks, based on their experiences. They acknowledge the complexity of decisions concerning CHW roles and tasks, noting that each decision affects the effectiveness, acceptability, feasibility, and costs associated with their roles. Scott *et al*. [36] described in their review of CHW programs that fostering respectful collaboration and communication between CHWs and higher-level staff can enable the health system to benefit from CHWs’ unique, practical knowledge. This integration enhances the acceptability and credibility of CHW-led programs.

Another important finding of this study relates to the pivotal role of CHWs in translating health information into meaningful, actionable, and sustainable health practices within communities. Given their close contact with community members and their own embeddedness in the community, CHWs possess invaluable insights into the cultural nuances that influence health behaviors and participation in health interventions. This insight enabled CHWs to adapt the program’s delivery in real time, tailoring language, incorporating resources, and providing culturally resonant examples that enhanced participant engagement and learning. Their collective efforts to revise and enhance program activities for greater interactivity and relevance triggered participant engagement in the intervention delivery, further affirming the indispensable role of CHWs in bridging the gap between health interventions and community needs. Findings underscore the benefits of a participatory approach, from design to implementation, leveraging the unique insights of CHWs to ensure health interventions are culturally sensitive, thereby fostering greater community engagement and program effectiveness. This observation is supported by Coulter *et al*. [37] in their scoping review on the role of CHWs within health intervention research teams. They documented specific benefits arising from the inclusion of CHWs as partners in health intervention research, such as validating study findings through their shared experiences with the population, and enhancing the potential for the intervention’s practical application and sustainability.

Our findings emphasize the profound impact of health interventions not only on the community but also on the CHWs who deliver them. This underscores the dual benefit of such programs, where CHWs, as integral members of the community they serve, gain personal health benefits while imparting knowledge to participants. CHWs’ narratives of personal change, from altering eating habits to adopting more active lifestyles and developing mindfulness techniques, highlight the intrinsic value of their participation in the intervention. The positive changes CHWs experienced in their physical health, emotional resilience, and approach to client interactions demonstrate the symbiotic relationship between teaching health principles and practicing them. This realization not only bolsters their credibility and effectiveness in the eyes of the community but also fosters a broader culture of health. Florindo *et al*. [38] present evidence that CHWs who maintain active lifestyles and healthy diets are more likely to counsel patients on the benefits of physical activity and healthy eating, highlighting the direct link between their personal health behaviors and their effectiveness in promoting health within their communities. Laurenzi *et al*. [39] discuss how CHWs navigate the intersection of their personal and professional lives, finding ways to connect personally while maintaining professional credibility. Preston *et al*. [40] suggest a framework aimed at improving the overall well-being and sustainable performance of the public health workforce, advocating for a cultural shift toward prioritizing their resilience and reducing stress. Similarly, Søvold *et al*. [41] underscore the critical global public health need to focus on the well-being of healthcare workers. They argue that self-care is essential for managing the demands and workload of their roles, aiding in achieving a better work-life balance, and safeguarding their health, well-being, and job and life satisfaction.

Any generalization of the findings from this study should be approached with caution. Although the intervention has the potential to be disseminated and scaled, especially across rural cities in southern Brazil, the diversity of this large country, the complexity of urban settings, and the variation in utilization of CHWs within primary healthcare will limit the degree to which our findings can be generalized. Another limitation is that the study period does not include initiatives carried out after 2017 that may have increased the qualification of health care teams.

In conclusion, our study reveals an important shift in community perceptions of CHWs driven by a behavioral change intervention. This shift challenges traditional healthcare paradigms by prioritizing wellness and self-care, dismantling established healthcare hierarchies, and fostering trust and identity among CHWs. The approach, characterized by cultural humility, plays a pivotal role in connecting health interventions with community needs. The personal health gains underscore the importance of self-prioritization for CHWs. Our research advocates for the implementation of programs and the revision of policies to further recognize and enhance the role of CHWs. The vital role of CHWs in primary healthcare and their essential contribution as partners in health promotion actions must be actively communicated to health officials and decision-makers to ensure their support and integration. These efforts are crucial for achieving significant improvements in public health and for advancing implementation science.
